# Anti-Corrosion Reinforcements Using Coating Technologies—A Review

**DOI:** 10.3390/polym14214782

**Published:** 2022-11-07

**Authors:** Lei Yan, Wenjie Deng, Neng Wang, Xuanyi Xue, Jianmin Hua, Zengshun Chen

**Affiliations:** 1 State Key Laboratory of Mountain Bridge and Tunnel Engineering, Chongqing Jiaotong University, Chongqing 400074, China; 2 School of Civil Engineering, Chongqing Three Gorges University, Chongqing 404100, China; 3 School of Civil Engineering, Chongqing University, Chongqing 400045, China; 4 School of Management Science and Real Estate, Chongqing University, Chongqing 400045, China

**Keywords:** anti-corrosion, corrosion epoxy coated reinforcement, galvanized reinforcement, stainless cladding steel reinforcement, steel-fiber reinforced polymer composite bar, fiber reinforced polymer

## Abstract

**Highlights:**

**What are the main findings?**

- Anti-corrosion reinforcements, including galvanized reinforcements, epoxy coated reinforcements, stainless cladding reinforcements and steel-fiber reinforced polymer composite bars, are reviewed.
- The impacts of the manufacturing process and material properties on the performance of anti-corrosion reinforcements are reviewed.
- The behaviors of anti-corrosion reinforcements, with and without corrosion, are described in detail.
- The development and application of anti-corrosion reinforcements are introduced.

**What is the implication of the main finding?**

- The anti-corrosion reinforcements conform to the development of modern structures.

**Abstract:**

Coated reinforcements are expected to improve the performance of reinforced concrete in aggressive environments, but different kinds of coated reinforcements can express a variety of properties, which can confuse researchers and engineers. This paper reviews the manufacture, corrosion mechanisms, behaviors, and applications of popular or promising coated reinforcements, incorporating galvanized reinforcements (GRs), epoxy coated reinforcements (ECRs), stainless cladding reinforcements (SCRs), and steel-fiber reinforced polymer composite bars (SFCBs). In terms of manufacture, GRs and ECRs should focus on minimizing the negative effect of manufacture on performance, while SCRs and SFCBs should reduce the cost and increase the production capacity. Behaviors of GRs and ECRs are primarily determined by the steel substrate, but the behaviors of SCRs and SFCBs are primarily affected by the coat and core, and their interaction. The corrosion mechanism of GRs and SCRs is about oxidation, while that of SFCBs is about hydrolysis. ECRs are usually corroded under film, which can be a cause of premature failure. Corrosion embrittles SCRs, as well as bare bars, but corrosion of SFCBs usually causes a reduction in maximum strength. The investigation of the corrosion behaviors of GRs and ECRs focuses on bond strength. GRs have controversial performance. ECRs have been proven to have drawbacks regarding bond strength. The use of anti-corrosion reinforcement is uneven in regions, which may correlate with the development of technology and the economy.

## 1. Introduction

Concrete is one of the most common materials for construction in the world [1]. Its strong compressive strength and economic cost offer a solution for most structural requirements. To enhance tensile ability and serviceability, concrete is always reinforced with steel reinforcements [2]. The good interaction between steel and concrete ensures that reinforced concrete exhibits the advantages of both. However, as detection of corrosive damages to construction built in the last century has become increasingly apparent, the challenges of dealing with corrosion require addressing [3]. Corrosion not only degrades the strength and ductility of the reinforcements, but also reduces the fatigue performance and bond strength. Thereby, the mechanical performance and serviceability of structures are compromised, typical signs of which include intensive cracks, large deflection, and even brittle failure. In the USA, the annual direct corrosion cost of highway bridges alone was about $8.3 billion in 2014 [4]. The American Society of Civil Engineering predicts that infrastructural repair will cost about $2 trillion by 2025 [5]. In China, the cost of reinforcement corrosion is about 1.2% of its Gross Domestic Product, which exceeded $2 trillion in 2021 [6]. 

To prevent corrosion in reinforced concrete structures, several practical applications are available, including concrete protective coatings, corrosion inhibitors, cathodic protection, and anti-corrosion reinforcement. Concrete protective coating enhances the civil life of a structure by blocking the penetration of detrimental elements, but the duration of effective protection is still less than the design lifetime of the structure [7,8,9,10]. The use of corrosion inhibitors could be a solution, but they may degrade the mechanical properties of concrete [11,12,13]. Cathodic protection is an effective method; however, it is expensive and difficult to install in some scenarios [2,14,15]. Anti-corrosion reinforcements are recognized as effective and practical ways to expand the lifetimes of structures in either normal or aggressive environments [8,16,17]. 

**Figure 1:**
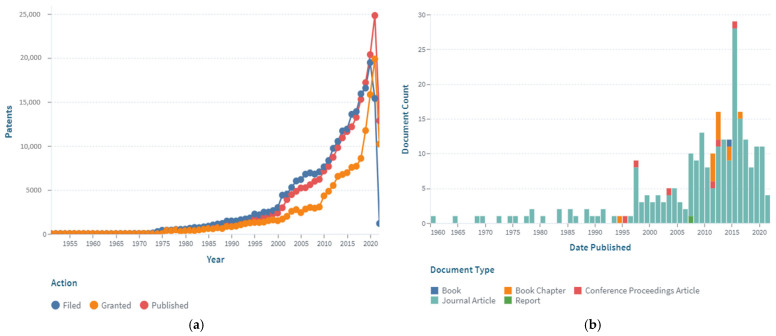
The publication analysis on anti-corrosion: (**a**) patents and (**b**) cited works in recent years [51,52].

**Figure 2:**
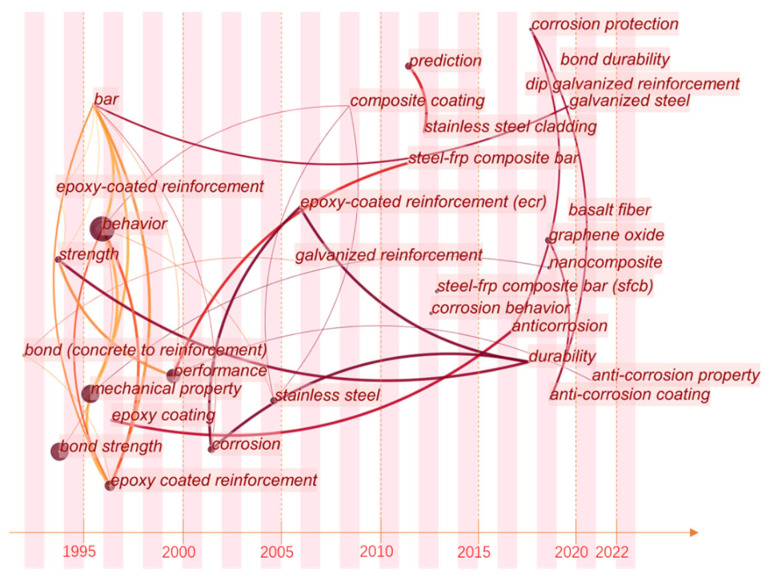
Combined frequency and centrality of keywords (the size of cycle and width of line represent the frequency and centrality).

As shown in Figure 1, there are numerous publications on anti-corrosion. Coating technology is a common way to resist corrosion [18,19,20,21,22]. In this paper, the research hot spots in the field of anti-corrosion reinforcements using coating technologies were studied by bibliometric analysis. Papers that contained the keywords “galvanized reinforcement”, “epoxy coated reinforcement”, “stainless cladding reinforcement”, and “steel-FRP composite bar” were extracted from the Web of Science Core Collection. The open-source software program Citespace 5.8.R3 was used to analyze data and visualize results [23,24,25,26]. As shown in Figure 2, papers written earlier mainly focused on mechanical properties and behaviors of GRs and ECRs without corrosion, and bond strength was highly emphasized. Since 2000, when anti-corrosion coatings and composite bars (such as SCRs and SFCBs) were invented, studies about corrosion behaviors began to emerge. Thus, this paper introduces the mechanical performance (tensile, fatigue, and bond behaviors) of GRs, ECRs, SCRs, and SFCBs, with particular emphasis on the effects of manufacture and corrosion. Then, applications and future works are addressed. 

**Figure 3:**
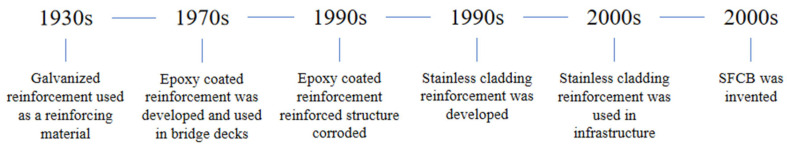
The development of anti-corrosion reinforcements using coating technologies [3,27,28,29,30,31,32,33,34,35,36,37,38,39,40,41,42,43].

The development of the review on reinforcements is shown in Figure 3. Galvanized reinforcement (GR) has been applied to construction since the 1930s [27], in which zinc serves as the coating, providing cathodic protection for steel. In the USA, de-icing salt was widely used in highway bridges in the 1960s, since bridge decks demand higher corrosion resistance at an acceptable cost [28]. Epoxy coated reinforcement (ECR) was developed and used in highway constructions in the 1970s [3,27,28,29]. However, as more cases of corroded epoxy coated reinforcements have been reported since the 1990s [3,28,30,31], the protection effectiveness of the epoxy coat has become controversial. To further improve the anti-corrosion and mechanical performance of reinforcements, stainless cladding reinforcement (SCR) and steel-FRP composite bars (SFCBs) were introduced in the 1990s and 2000s [32,33,34,35,36,37,38,39,40,41], respectively. These reinforcements were founded on the reliable corrosion resistance of stainless steel and FRP. In practice, SCR has been adopted in certain infrastructures since the 2000s [42,43], but the application of SFCBs remains deficient.

Current reviews study novel methods to enhance corrosion resistance or focus on one type of anti-corrosion coating and its application [18,19,20,21,22,44,45,46,47,48,49,50]. Specifically, Ding [48] reviewed the improvements offered by using graphene in organic anti-corrosion coatings. Lazorenko et al. [49] reviewed anti-corrosion coatings, including organic, inorganic, metallic and combined coatings, in the protection of steel railway structures from atmospheric corrosion. In terms of coated reinforcements, Zemajtis [28] reviewed the performance of bridges reinforced by epoxy coated reinforcements. Yeomans [16] presented an overview of galvanized reinforcements. Sun et al. [50] summarized the behaviors of SFCBs and structures reinforced by SFCBs. However, SCRs have not been reviewed, even though they have had wide applications. Furthermore, there are only rare reviews comparing the mechanical properties and behaviors, with and without corrosion, of multiple anti-corrosion coating technologies. Yet a comparison is necessary for the popularization and development of anti-corrosion reinforcements using coating technologies. Thus, this review aimed to comprehensively compare the most popular coating technologies that apply to reinforcements and to explore the value of anti-corrosion reinforcements in civil construction.

## 2. Manufacture

Over the past century, GRs have been widely investigated as an alternative coating to protect steel from corrosion [16,53]. GR is generally formed by having a zinc layer, metallurgical layer, and base steel. There are varying ways to coat zinc on the surface of the steel, one of which, the hot-dip method, is recognized as being an economical technology [2]. As shown in Table 1, zinc has lower strength and elastic modulus than steel, and, regarding coating thickness, the zinc layer is usually thin. Thus, the steel substrate dominates the tensile properties of bars. The hot-dip method has negligible influence on the mechanical properties of normal and low strength grade bars because the bars have not been excessively cold worked during fabrication. High strength bars may exhibit slight improvements in strength and ductility, due to minor stress relief. Cold-twisted steel bars can be embrittled by the hot-dip method [53,54]. 

The manufacture of epoxy coating on bars includes methods such as spray-on, dipped-in, shelling, and electrostatic spraying; the most common of which is electrostatic spraying [55,56]. As shown in Table 1, epoxy coating generally has an insignificant influence on the basic mechanical property of reinforcement, due to its low strength, stiffness and minor thickness. However, an early report pointed out that the epoxy coating may slightly embrittle the reinforcement [31]. 

Since the 1990s, researchers have shown an increasing interest in SCRs [33,34,35]. The behaviors of SCRs are affected by cladding and core, and their interaction. Since the properties of stainless steel and steel have similarities, which are shown in Table 1, their interaction becomes an important issue to ensure the composite action of the two materials. Hot-rolling is an effective technology to achieve proper interaction by forming a metallurgical bond between steel and stainless steel [33,57,58,59]. With increase in rolling temperature, the promotion of strength becomes more distinct [58]. However, early hot-rolling SCR still had problems of insufficient rib height, de-bonding between the coating and core, and expensive costs [32,60]. In 2015, Hunan 3T New Materials CO., LTD., produced the stainless-clad bimetallic steel bar (SCBSB) by continuous hot rolling with a net interfacial compound [60]. The corrosion resistance and mechanical properties of SCRs were guaranteed.

SFCB improves the performance of steel reinforcement by winding fibers around the rebar [36,37,61]. The fiber used is well-known for its use in FRP. Technically, the rust and longitude ribs of the steel substrate are removed. Then, the bar is wound by the roving that fills the gaps between the ribs. The above measures ensure that the FRP layer bonds well with the steel bar. Unlike previous coated bars, the protective coat of SFCBs plays a distinct part in the tensile behavior of reinforcement because of having higher strength and thicker coating than GRs and ECRs, as shown in Table 1 [36,50,62,63]. The FRP coat can be made from carbon fiber reinforced polymer (CFRP), glass fiber reinforced polymer (GFRP), or basalt fiber reinforced polymer (BFRP). BFRP is mainly recommended [36,62,63,64].

**Table 1:** Properties of components of coated reinforcements.

Item	Elastic Modulus (GPa)	Yield Strength (MPa)	Tensile Strength (MPa)	Density (g/cm^3^)	Elongation Rate (%)	Coating Thickness (mm)	Refs.
BSR	200	276~517	483~690	7.85	6~12	-	[65,66]
Zinc	108	75	-	7.14	-	0.085~0.087	[16,67]
Epoxy	22~35	-	100~220	1.2	-	0.13~0.23	[3,30]
Basalt fiber (CBF13)	90	-	2250	2.63	2.5	2.1~3.4	[36]
Resin (Atlac 430)	3.6	-	95	1.06	6.1	-	[36]
S30408	204	>205	520	7.93	35	1.7~3.1	[33,68]

## 3. Behavior without Corrosion

### 3.1. Tensile Properties

**Figure 4:**
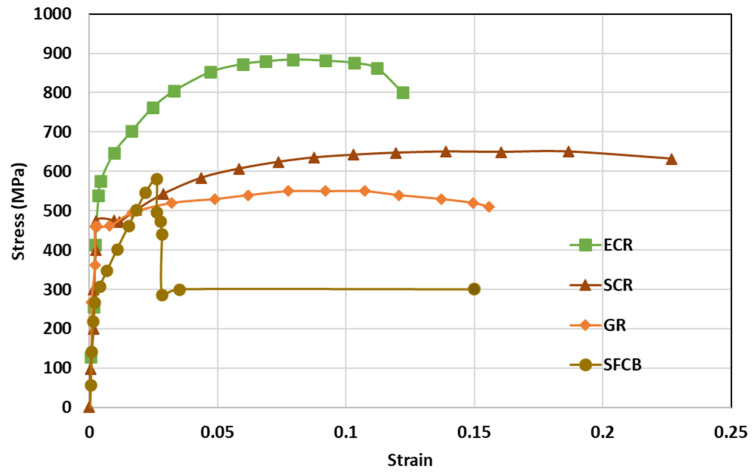
Comparison of typical tensile properties of GR [70], ECR [27], SCR [74], and SFCB [75].

The tensile properties of anti-corrosion reinforcements are summarized in Table 2, and Figure 4 draws the typical strain–stress curve of anti-corrosion reinforcements. In general, the elastic modulus, ductility, and failure modes were compared, but the values of yield strengths and ultimate strengths are not discussed here, because different steels are adopted. Specifically, galvanized reinforcements (GRs), epoxy coated reinforcements (ECRs), and stainless cladding reinforcements (SCRs) have similar elastic modulus and failure mode to black steel reinforcements (BSRs), where ductile fracture is usually observed. The stress of SFCB continues to increase linearly after steel yielding because of the high strength of the fiber. There is a sudden drop of stress after fiber rupture but the steel prevents full stress dissipation. As shown in Table 2, the coating and core of SFCBs show different failure behaviors, which are blow-out fracture and ductile fracture, respectively. In contrast, SCRs show ductile fracture, and stainless steel cladding and steel substrate still bond well after the has been SCR necked. SCR has enhanced ductility, over other reinforcements, because stainless steel is more ductile than black steel.

**Table 2:** Tensile properties of reinforcements.

Item	Elastic Modulus (GPa)	Elongation	Failure Mode	Refs.
BSR	200	0.06~0.12	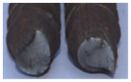	[65,66,69]
GR	192	0.11~0.16	Similar to bare bar, steel substrate dominates the failure mode.	[27,54,70]
ECR	192	0.11~0.14	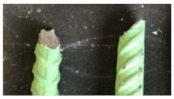	[27,31,71]
SCR	185~196	0.22~0.24	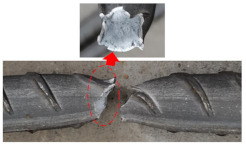	[33,57,60,72,73]
SFCB	123~168	0.15	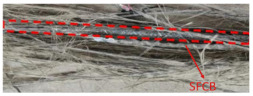	[36,37,39,62,63]

Note: black steel reinforcement (BSR); galvanized reinforcement (GR); epoxy coated reinforcement (ECR); stainless cladding reinforcement (SCR); steel-FRP composite bar (SFCB).

### 3.2. Fatigue Properties 

In coastal constructions, seismic performance is always considered because plate activity is relatively active in coastal areas. Coastal areas also need anti-corrosion reinforcements. As confirmed by Sheng and Gong [76], stress events caused by earthquakes are those of low-cycle fatigue. Investigations of GRs and ECRs are deficient. The reason could be that galvanized coating and epoxy coating rarely participate in the mechanical properties of reinforcements. Current investigations of fatigue in SCRs and SFCBs are shown in Table 3. The failure mode of tubby SFCBs is fiber fracture with fiber split, while that of slender SFCBs is mainly fiber split. More energy is dissipated in the tensile zone than in the compression zone, corresponding to the anisotropy of SFCBs. Bidirectional degradation effect is found after initial fiber failure. The fatigue performance of SFCBs is dominated by the fiber layer [75,77]. Yang et al. [78] studied the fatigue performance of ballastless track slabs subjected to different fatigue loading. Their results showed that after 3,000,000 fatigue cycles, the crack width of the SFCB slab was only 0.15 mm. At higher load levels, the traditional RC slab was close to fracture, while the stiffness of SFCB only reduced by 6%.

In terms of SCRs, the failure mode of SCRs in fatigue is buckling, and the buckling deformation is more apparent with increase in the strain amplitude. The energy dissipations in compression and tension zones are approximately equal, which corresponds to the isotropy of steel. The fatigue performance of the SCR was close to the BSR in [69,79,80]. Besides seismic performance, fire-resistance is also an important criterion in construction [81]. The basic post-fire mechanical properties of reinforcements have been widely investigated [74,82,83,84,85,86,87], but there are scarce resources for post-fire low-cycle fatigue properties [88,89]. Hua et al. [90,91] tested the low-cycle fatigue properties of SCRs with different elevated temperatures and cooling methods. Specifically, the SCR kept metallurgically bonding after fatigue or post-fire fatigue. The elevated temperature could cause a reduction in the number of cycles leading to fatigue. When the temperature was above 800 °C, the energy dissipation per cycle experienced a dramatic drop, which indicated that the elasticity of the SCR suddenly decreased. Temperature had little effect on the hysteresis curves for SCR subjected to cooling in air, but the curves of SCR subjected to cooling in water became slender when temperature exceeded 700 °C.

**Table 3:** Experiments on fatigue properties.

Ref	Type	Variable	Remarks			
Sun et al., 2017 [75]	BSR/ SFCB	Bar type	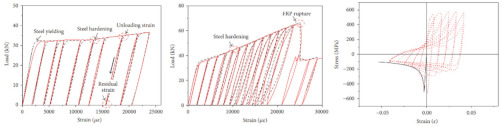			
Li et al., 2022 [69]	SCR	Diameter, strain rate, strain amplitude	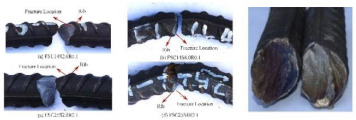			
Hua et al., 2022 [79]	SCR	Strain amplitudes	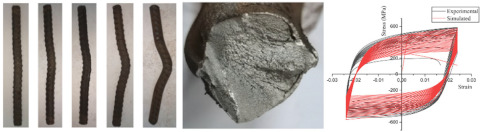			
Wang et al., 2022 [80]	SCR	Slenderness ratio, fatigue strain	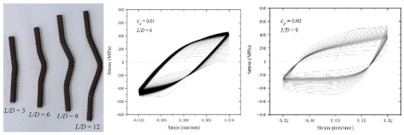			
Hua et al., 2022 [92]	SCR	fatigue damage	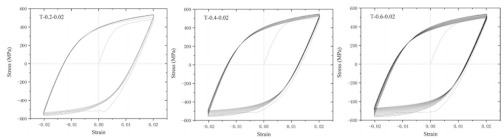			
Hua et al., 2021 [90]	SCR	Exposure temperature	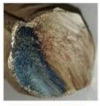 25 °C	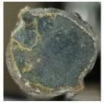 900 °C	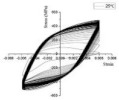 25 °C	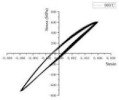 900 °C
Hua et al., 2022 [91]	SCR	Exposure temperature, cooling method	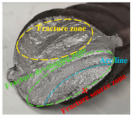 700 °C and CIW	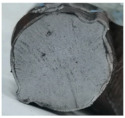 700 °C and CIA	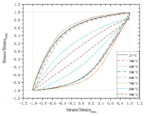 CIW	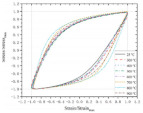 CIA

Note: stainless cladding reinforcement (SCR); steel-FRP composite bar (SFCB); cooling in water (CIW); cooling in air (CIA).

### 3.3. Bond Strength

As the baseline, the bond strengths of steel bars have been commonly tested [93,94,95,96]. Long et al. [93] concluded that shallow embedment length was a cause of pull-out failure, where the load–displacement curve had a hump and nearly constant residual force. In this situation, the specimen failed before steel yielding. Increasing the embedment length could reduce the hazard of slipping failure, where the load–displacement curve showed strength development in the post-yield stage. Furthermore, with increase in loading rate, bond strength developed. The reason for this could be that radial cracks were limited to propagating to concrete by a higher loading rate.

Certain investigators have reported a reduction of bond strength by using GRs [27,95,97,98]. These experiments included pull-out, beam, and beam to column. The bond strength of GRs to concrete experienced a reduction of 4 to 17%. Pokorný [95] attributed the drop in bond strength to oxidizing zinc and hydrogen formation. However, in a beam end test, Kayali [96] reported that the bond strength of GRs fairly equaled steel bars, and further surface treatment was not necessary for the GR. Patnaik [27] proposed the pull-out test for BSRs and GRs. According to Patnaik’s results, the GR showed higher bond strength than the BSR, with 17% enhancement. 

Many recent studies [27,31,96,99,100] have shown that ECR is deficient in bond strength, with the reduction varying from 5 to 35%. A larger decrease in bond strength was observed in beam tests in [31,99,100] than in pull-out tests in [27,96]. ECR reinforced concrete not only has lower bond strength, but the slipping distance at the same bond stress is also larger than that of uncoated bar reinforced concrete. Hasan et al. [31] pointed out that the concrete cover to the bar diameter ratio and the rib bearing area ratio were explicitly associated with bond strength. In beam tests, the thick concrete cover and strong rib indicated better bond performance. Furthermore, Assaad and Issa [100] concluded that the adhesion loss and relatively smooth surface of epoxy coating could be the cause of the decrease in bond strength. 

There is a renewal of bond strength between SCRs and sea-sand concrete [101,102]. As concluded by Hua et al. [101], there was no obvious damage to SCRs after specimen failure. The specimens with longer concrete age had better bond performance. Increasing the concrete cover thickness to bar diameter ratio and adding polyoxymethylene fiber could strengthen the bond between SCRs with sea-sand concrete. 

Although SFCBs improve the mechanical behavior of FRP bars, the bond strength of SFCBs still needs to be developed [94]. Surface-threaded treatment could develop the bond performance of SFCBs [94]. Similar to the failure mode of ribbed BSRs, surface-threaded SFCBs experience splitting failure, which indicates that the stress can properly transfer to concrete. The application of a sand coat was regarded as a method to develop the bond strength of FRP bars by increasing surface roughness. The bond strength of sand-coated SFCBs were enhanced to a maximum of 50% over that of SFCBs without a sand coating [62].

**Figure 5:**
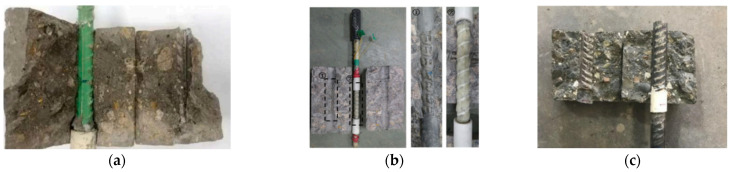
Failure performances of (**a**) ECR [105], (**b**) SFCB [94], (**c**) SCR in the bond test [101].

**Figure 6:**
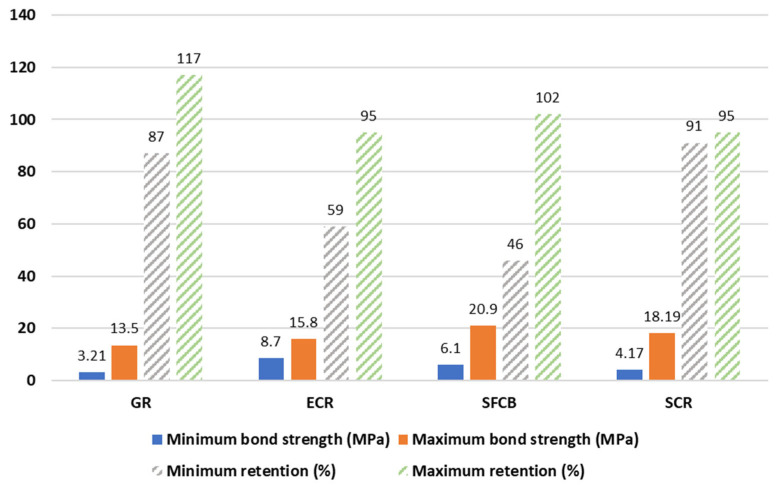
Bond strengths and bond strength retentions due to corrosion [39,94,95,96,98,102].

Table 4 summarizes the bond strength tests for BSRs, ECRs, GRs, and SFCBs. The retention is defined as the bond strength of anti-corrosion reinforcement divided by the bond strength of BSRs. Specifically, most of the tests indicate that existing coat technologies have drawbacks in relation to bond strength. Apart from the effects of cover thickness and bar diameter, the reasons for reduction of bond strength can include the following: low stiffness, poor toughness, adhesion loss between the coat and bar, and deficient strength of the rib. Surface threading and sand coating can improve the bond performance of SFCBs, but brittle failures occurred in sand-coated GFRP specimens in pull-out tests in [62]. Figure 5 shows examples of failure performance. Specifically, the ribs of ECRs are worn-out after pull-out progress, while the resin surface of SFCBs is also scratched. In contrast, SCRs shear off the concrete between ribs. This diversity is caused by different strengths of coating. Figure 6 compares the bond strengths and bond strength retentions of anti-corrosion reinforcements. The bond strength of anti-corrosion reinforcements is distributed in the range from 3.21 to 20.9Mpa. The GR, ECR, and SFCB show deficient bond strength retention at lower bounds. Nevertheless, the bond strength retention of ECRs is still less than 100% at the upper bound. The diversity of bond strength retention reflects the contradiction of GRs investigations but proves the shortage of ECRs. The bond strength of SCRs is close to that of BSRs at the upper and lower bounds. This indicates that the performance of bond strength in SCRs is similar to BSRs. The SFCB satisfies bond strength at the upper bond because it adopts the surface-threaded and sand-coated techniques. 

**Table 4:** Experiments on bond strength.

Bar	Test	Bond Strength (MPa)	Retention %	Ref.
BSR	Pull-out	18.8~22.6	-	[93]
BSR	Beams	3.34~3.84	-	[95]
BSR	Beam end	13.2	-	[96]
BSR	Pull-out	12.9	-	[94]
ECR	Pull-out	-	93	[27]
ECR	Beams	-	65~85	[99]
ECR	Beams	-	59~95	[31]
ECR	Pull-out	8.7~15.8	81~89	[100]
ECR	Beam end	9.0~9.9	68~75	[96]
GR	Pull-out	-	117	[27]
GR	Beams	3.21~3.61	94~96	[95]
GR	Beam-column	-	83~94	[97]
GR	Beam end	13.5	102	[96]
GR	Pull-out	13.1	87	[98]
SCR	Pull-out	4.17~18.19	-	[101]
SCR	Pull-out	9.4~16.5	91~95	[102]
SFCB	Pull-out	6.1~13.5	46~102	[94]
SFCB	Pull-out	11.6~15.5	-	[62]
SFCB	Pull-out	20.1~20.9	97~101	[39]
SFCB	Pull-out	11.0~20.9	65	[103]
SFCB	Pull-out	11.8~16.9	59~95	[104]

Note: black steel reinforcement (BSR); galvanized reinforcement (GR); epoxy coated reinforcement (ECR); stainless cladding reinforcement (SCR); steel-FRP composite bar (SFCB).

## 4. Behavior after Corrosion

### 4.1. Corrosion Mechanism

**Figure 7:**
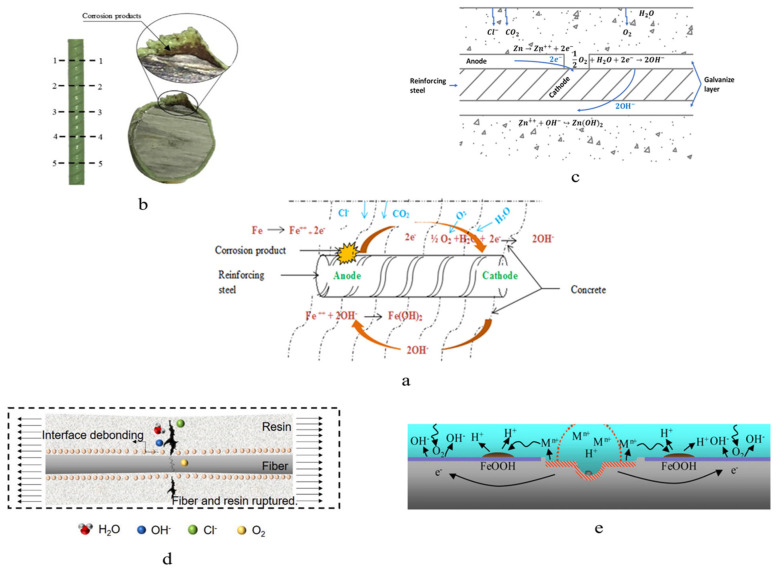
Corrosion mechanism of the (**a**) BSR, (**b**) ECR, (**c**) GR, (**d**) FRP bar, (**e**) Stainless reinforcement [2,30,111,123,128].

The corrosion mechanism can be classified according to material types, namely metal and nonmetal. For metal materials, oxidation is a common challenge. The metal can generate a passive film to prevent corrosion. The passive layer of black steel reinforcements (BSRs) is a submicroscopic γ-Fe_2_O_3_ film [12,106]. However, it can be attacked by Cl^−^ [8,106,107,108]. The chloride ions can replace the oxygen ions [106,107,108], and the insoluble oxide is, thus, transmuted to soluble chlorine salt. After the passive film is damaged, the steel bar is electrochemically corroded. As shown in Figure 7a, the cathodic and anodic reactions occur simultaneously at the surface of BSRs where the steel matrix transfers the ions [8,109]. The iron atoms lose electrons to the anode. The ferrous ions combine with free hydroxide ions, which produces ferrous hydroxide. The ferrous hydroxide is further oxidized to ferric oxide, generally called rust [109]. In consequence, rust that is 2–10 larger than the passive product gathers on bars, which expands the concrete by a pressure of 3–4 MPa [16]. A crack thereby propagates from the bars to the surface of the concrete. In terms of galvanized reinforcements (GRs) and stainless cladding reinforcements (SCRs), either zinc or stainless steel can generate a more stable passive film than steel, which are Zn-based and Cr-based oxides, respectively. The galvanized coat can provide extra protection as the sacrificed coat [110]. As shown in Figure 7c, in the case of the steel partly exposed to an aggressive environment, the zinc loses electrons instead of the iron. The oxidation of zinc is dense and steady, and further corrosion can be prevented [8,111]. For SCRs, the corrosion mechanism of stainless steel is a complex subject that is worth research driven by practical significance. Pit corrosion is the important mechanism in stainless reinforcement in the marine environment. As shown in Figure 7e, the mechanism of pitting corrosion is a negative feedback loop of local acidification [112]. Apart from chemical corrosion, corrosion under sustained stress is also worth investigating when stainless steel is adopted as the reinforcement [113,114]. The elastic modulus of most stainless steel is around 200 GPa, but the elastic modulus of Cr_2_O_3_ is 280 GPa at 25 ℃ [115]. Due to this difference, the passive film can physically rupture when deformation occurs. In terms of non-metal material, moisture is the cause of degradation. For example, as an organic material, epoxy has certain permeability which allows the passage of corrosion substances [110,116]. Once enough corrosion products accumulate on the surface of steel, disbondment is accelerated because of the expansion stress from rust and blisters [110,116]. The schematic of under-film corrosion is shown in Figure 7b [30]. As a recent research hot spot, graphene oxide nanocomposite was found effective in improving the corrosion resistance of epoxy coating, as shown in Figure 3 [117,118,119,120]. The FRP (Fiber Reinforced Polymer) coat principally accounts for the corrosion resistance of SFCBs [38]. FRPs can be decomposed by strong alkaline conditions in a wet environment, which is the basis of hydrolysis [40,121,122,123,124]. As shown in Figure 7d, the fiber and resin de-bond in hydrolysis. Subjected to sustained load, corrosion channels appear and promote moisture diffusion. These damages shorten creep rupture time [40,123,124]. The chloride threshold is the concentration of chloride on the surface of reinforcement required to decompose the passive layer [125,126,127]. It is one of the parameters to describe the corrosion resistance of reinforcements. The second Strategic Highway Research Program (SHRP2) summarized the chloride threshold for various reinforcement steel types. The average chloride threshold of BSRs was 0.68; while the average chloride thresholds of GRs, ECRs, and SCRs were1.27, 0.63, and 5.2 [125]. The chloride threshold of SFCBs was not reported. ECRs have a lower average chloride threshold than BSRs, attributed to it being difficult to avoid the coating defect in the field. 

### 4.2. Tensile Strength

In terms of the mechanical properties of steel bars after corrosion, a large number of experiments have been conducted [106,129,130,131]. Xu et al. [129] reported that the yield plateau was observed to shorten and even disappear when the mass loss was more than 2%. As reported by C.A. Apostolopoulos et al. [130,132], both effective and apparent strength decreased with an increase in exposed days, but apparent strength experienced a more dramatic drop. Almusallam [106] also concluded that the elongation showed distinct degradation with corrosion. Zhang et al. [131] reported that the ultimate strength closed to yield strength with aggravating corrosion, which implied a higher risk of brittle failure. 

Investigations into the mechanical properties of coated bars after corrosion have been more recent [33,39,70,133,134]. Ismail and Muhammad [70] tested the tensile behavior of corroded GRs. The zinc was depleted in corrosion, but the yield strength, ultimate strength, and elongation to fracture were observed to develop after immersion. Miyazato and Nakazawa [135] experimented on the tensile performance of ECR corresponding to varying damage areas. Their results showed that tensile strength was basically maintained under a chloride environment. Gao [133] tested the mechanical behavior of SFCBs subjected to corrosion and sustained load. The ultimate strength of SFCBs was observed to decrease after immersion, with strength retention of 88.84%. Ge et al. [39] presented a similar test and found the maximum strength retention was 87.27%. Zhou et al. [136] polarized the CFRP bar and SFCB to assess the feasibility of the bars with impressed current cathodic protection (ICCP). In the result, based on the prediction model, the service life of the CFRP bar was 62 years, while that of SFCB was only 7 years. The reason was that the inner steel of SFCB corrodes much faster than the resin of CFRP. Zhou et al. [137] reported the corrosion rate of carbon-type SFCB was less than 1/10 of BSR, and that of glass-type SFCB was less than 1/100 of BSR. Hua et al. conducted electrochemical accelerated corrosion tests for SCRs [33,134,138] and reported that both apparent yield strength and ultimate strength of SCRs decreased after corrosion, but the reduction of yield strength was less than that of BSR. They also proposed predictive equations and a stress–strain model to clarify the corrosion performances and mechanical properties of artificially damaged SCRs with different corrosion ratios.

**Figure 8:**
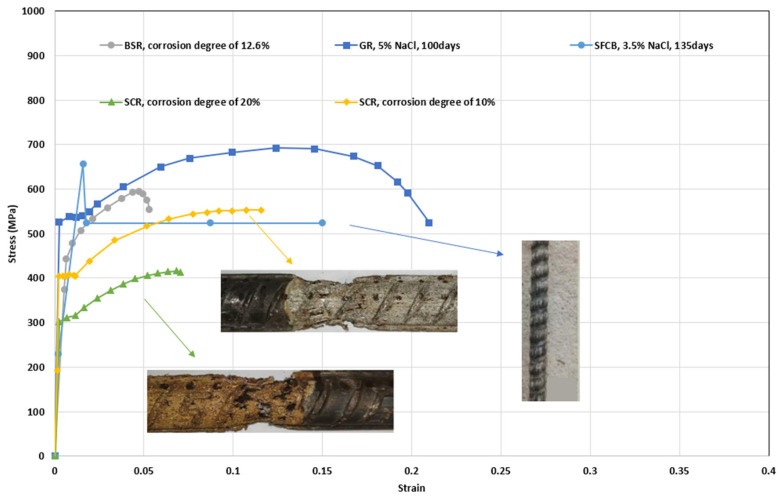
Tensile behaviors of BSR [106], GR [70], SFCB [39], and SCR [33] after corrosion.

Table 5 summarizes the experiments on the tensile behavior of corroded bars. The experimental results generally concern retention of yield strength, ultimate strength, and elongation to fracture. A part of the investigation compares the results to current specifications. Specifically, BSRs have been widely studied [106,129,130] and the common drawback reported is the rapid degradation of elongation to fracture in BSRs under corrosion. In terms of GRs and ECRs, there are scarce investigations on tensile strength. The decomposition of the galvanized layer has a negligible effect on the tensile strength of reinforcement [70]. The degradation of SFCBs is generally expressed as a reduction of maximum strength [39,133]. After corrosion, SCRs also show degradation of ductility, but the degree of degradation is lower than for BSRs [33,134]. Figure 8 compares the partial tensile behaviors of BSRs, GRs, SFCBs, and SCRs. Subjected to NaCl solution, the coatings of GRs and SFCBs corroded, but the steel substrate was effectively protected. The tensile behaviors of corroded specimens, thereby, had no obvious changes to the uncorroded specimens [39,70,133]. The corroded tensile behaviors of BSRs is expressed as embrittlement [106,130,131,132]. The yield plateau of BSRs disappears when the corrosion degree reaches 12.6% [106]. However, for SCRs, necking is still observed even when the corrosion degree reaches 20% [33]. This could be evidence that the degradation of SCRs is slower than that of BSRs under corrosion. 

**Table 5:** Accelerating tests of uncoated bars and coated reinforcements.

Ref.	Type	Conditions	Solution	Remarks
Almusallam 2001 [106]	BSR	Electronic corrosion in concrete	5% NaCl	Proved that the elongation has a distinct degradation with corrosion.
Ismail 1997 [70]	GR	Salt solution bath in concrete	5% NaCl	Found that the tensile properties are improved after immersion.
Miyazato [135]	ECR	Electronic corrosion	Chloride environment	Found that the speed of corrosion correlates to the damage area of ECR.
Gao 2020 [133]	SFCB	Solution bath with sustained stress	3.5, 7.0% NaCl	Found that the ultimate tensile strength is degraded.
Zhou et al., 2020 [136]	SFCB	Electronic corrosion	Seawater	Found that compared with CFRP, the deterioration of SFCB is serious due to the corrosion of the inner steel.
Ge et al., 2021 [39]	SFCB	Solution bath with sustained stress	3.5, 7.0% NaCl	Proved that the sustained stress amplifies the degradation.
Hua et al., 2021 [33]	SCR	Electronic corrosion	5% NaCl	Found that the corrosion has a negligible effect on yield strain.
Hua et al., 2021 [134]	SCR	Electronic corrosion	5% NaCl	Found that the *ε_u_* and *ε_u_/ε_y_* decrease with intensifying the corrosion.
Hua et al., 2021 [138]	SCR	Electronic corrosion	5% NaCl	Found that the increase of corrosion ratio led to the decrease of *f_y_*, *f_u_*, *ε_u_* and *δ*, and under the same corrosion ratio, the *f_y_* and *f_u_* of corroded BSRs increased with the increase of *W/D*.

Note: black steel reinforcement (BSR); galvanized reinforcement (GR); stainless cladding reinforcement (SCR); steel-FRP composite bar (SFCB).

### 4.3. Fatigue Properties

Hasegawa et al. [54] discussed the effect of humidity on the corrosion fatigue mechanism of galvanized AISI 1045 steel. In the low cycle fatigue region, the fatigue strength of galvanized specimens showed no obvious difference with change in humidity. However, a reduction of fatigue strength due to galvanization was reported when the number of cycles to failure was larger than 10^4^. Hua et al. [134,139] investigated the corroded low-cycle fatigue performance of SCRs. SCRs were electronically corroded to a controlled degree. The corrosion initialized at the surface of SCRs and gradually deepened. With increase of corrosion degree and fatigue strain amplitude, the cycles of fatigue and total energy dissipation reduced. The above investigations are compared in Table 6. Both reinforcements show ductile fractures in the low-cycle region. There are gaps in the interface of the galvanized layer and steel core, a separation that was also observed in the SCR when the strain amplitude increased [139]. 

So far, a deficiency of investigations in corroded tensile and fatigue properties of ECRs has been found. The basic reason is that the steel substrate dominates the tensile and fatigue properties of ECRs. Even when considering corrosion, researchers prefer to investigate corrosion resistance based on individual electrochemical theories or by experiments on individual components, but mechanical tests are not involved [140,141,142,143,144,145]. 

### 4.4. Bond Strength

**Figure 9:**
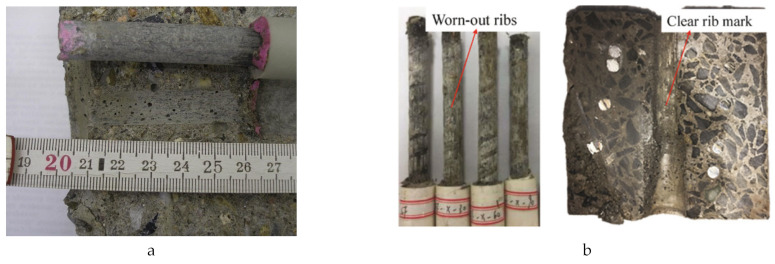
Failure performance of (**a**) GR and (**b**) SFCB in the pull-out test [2,38].

Corroded bond strength is a major area of interest within the field of coated reinforcement. The coat is commonly expected to improve the bond performance of reinforcement to concrete in an aggressive environment. In cases of galvanized coating, Pokorný et al. [2] pointed out that degradation of bond strength in GR reinforced concrete was mitigated. The evidence for this was found in research [146,147]. According to the results presented, the bond strength of galvanized specimens was usually less than that of black steel specimens without corrosion. However, after accelerating tests, this difference was almost eliminated, and the bond strength of galvanized specimens was even beyond that of the black steel specimens. Accumulation of hydrogen and decomposition of the zinc layer could be the reasons for the degradation of bond strength. As shown in Figure 9a, hydrogen gas is generated at the interface and increases porosity [2]. 

**Figure 10:**
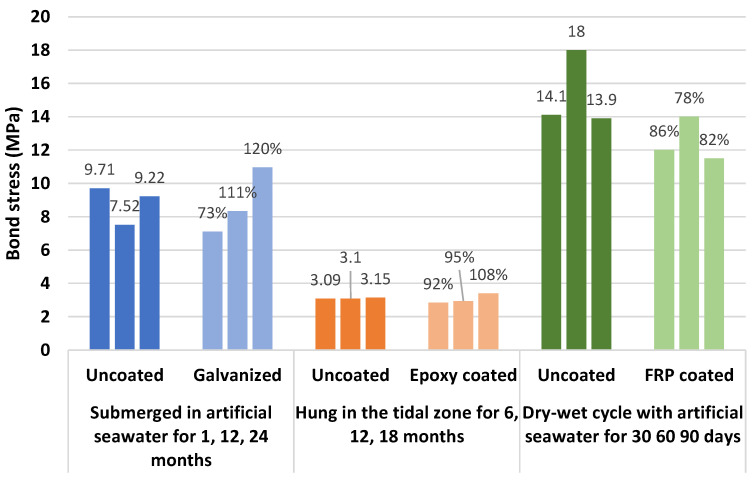
Bond strengths and retentions of uncoated and coated reinforcement exposed to varying environments [38,147,149].

Similarly, epoxy coats can also develop bond strength between concrete and reinforcement during corrosion [148]. El-Hawary [149] set specimens in tanks of seawater. With increase in exposure time, the bond strengths of the BSR reinforced specimens decreased faster than those of the ECR reinforced specimens. As shown in Figure 10, after 18 months of exposure in the Gulf, the bond strength in ECR reinforced specimens climbed to 108% of the bond strength in uncoated bar reinforced specimens. This enhancement was attributed to salt crystallization in the concrete pores. 

In terms of SFCBs, bond performance is different to that of previously coated reinforcement. The bond strength increases when SFCBs start to be exposed to an aggressive environment, then decreases with exposure time [38,39,150]. The swelling effect of FRPs in a wet environment could be a cause of the enhanced bond strength, which the corrosion degradation counteracts, exceeding the swelling effect, as shown in Figure 9b [150].

As shown in Figure 10, the bond strength of SFCB reinforced specimens is not sensitive to an exposed environment. Failure performances are similar before and after corrosion, comparing Figure 5c to Figure 7b. The enhancement of bond strength is ascribed to the swelling effect of the outer FRP in a water environment. In contrast, the bond strength of uncoated bar reinforced specimens increases at first because the corrosion products roughen the surface of the bar. Then, the bond strength drops due to the expansion stress of rust. Furthermore, SFCBs are reported to suffer more severe corrosion in conditions of continuous immersion, compared to a wet–dry cycling environment, while BSRs are the opposite [150]. 

Table 7 summarizes the tests of bond strength with corrosion, where retention is defined as the bond strength of coated bar reinforced specimens divided by bond strength of BSR reinforced specimens. Specifically, galvanized technology develops the bond strength of reinforcement and concrete with corrosion [27,146,147]. The epoxy coat is effective when exposed to an ocean environment [149]. The bond strength of SFCB reinforced concrete is controversial. It has good performance in a wet–dry environment [150]. However, corrosion may be severe when the specimens are continuously immersed in salt solution or seawater [39,150]. Further investigations are necessary. 

Combining Table 7 with Table 5 it can be deduced that acceleration tests usually simulate the aggressive environment by electrochemical corrosion, solution immersion, salt spray, and wet–dry cycle, and the relevant solutions include artificial seawater, natural seawater, 3.5% NaCl, corresponding to the marine environment, or 5%NaCl, corresponding to concrete pore fluid. The corrosion set depends on the experimental objectives and the corrosion resistance of reinforcements. For example, electrochemical corrosion was found in experiments interested in mechanical behaviors of metal reinforcements in varying corrosion degrees. Indeed, these experiments usually consider intensive corrosion. In contrast, non-electrochemical corrosion can be used in metal and nonmetal reinforcements, and the experiments also try to quantify the corroded performance of reinforcements on a time scale. In the anti-corrosion reinforcements that were reviewed, the experiments on GRs and ECRs included electrochemical and non-electrochemical corrosion. SFCBs were experimentally corroded by solution because their corrosion mechanism is close to that of FRPs. SCRs are only electrochemically corroded in experiments because it is difficult to simulate a corrosive solution for the concrete or natural environment that corrodes SCRs in an acceptable time frame. 

**Table 7:** Experiments on bond strength after corrosion.

Bar	Test	Condition	Solution	Period	Retention %	Ref.
GR	Pull-out	Electrochemical corrosion	pH 12 3.5% NaCl	6, 14, 18 days	105~115	[146]
GR	Pull-out	Electrochemical corrosion	5% NaCl	10 days	120	[27]
GR	Beam	Half immersion	Artificial seawater natural seawater	1, 12, 24, 60, 120 months.	73~120	[147]
ECR	Pull-out	Electrochemical corrosion	5% NaCl	10 days	87	[27]
ECR	Pull-out	Half immersion	Seawater	0, 6, 12,18 months	84~108	[149]
SFCB	Pull-out	Solution bath with sustained stress	3.5, 7.0% NaCl	45, 90, 135 days.	85~102	[39]
SFCB	Pull-out; Beam	Wet–dry cycles	Artificial seawater	30, 60, 90 days.	78~86	[38]
SFCB	Pull-out	Wet–dry cycles	Artificial seawater	30, 60, 90 days.	91~135	[150]
SFCB	Pull-out	Wet–dry cycles	Artificial seawater	3, 6, 9 months	74~99	[151]

Note: galvanized reinforcement (GR); epoxy coated reinforcement (ECR); steel-FRP composite bar (SFCB).

## 5. Applications

Over the past century, there has been an increase in the use of galvanized steel as a reinforcing material [27]. In the 1930s, civil construction in the USA regularly used GRs. In the 1950s, GRs started to be applied to bridge decks, which require strict crack control [27]. Yeomans [16] recorded field studies on galvanized steel reinforced bridges built from 1968 to 1979 in the USA and UK. The studies indicated that the galvanized coat offered effective protection for 15 to 18 years after the bridge was built. In terms of epoxy coated reinforcement, the application in bridges can be traced back to 1973 [27]. Epoxy coating was used to reinforce the substructures of a segmental bridge in the Florida Keys, USA. Since the 1970s, ECR has been widely used in bridges in the USA. However, certain field studies at the end of the 1990s reported that the epoxy coat lost efficiency in protecting steel from corrosion after 15 to 22 years of service [3,28]. The application of SCRs was mainly concentrated in the early 21st century [42,43]. 

In terms of cost, the galvanizing bar is twice as expensive as the steel bar [72], which could cause up to 50% extra initial construction costs, depending on the region [16]. GRs shows cost-effectiveness in life cycle analysis, compared to BSRs [53]. However, repair is still necessary because the durability of galvanizing reinforcement has not yet met the requirements of a 100-year design lifetime [8]. The effective protection period of the epoxy coat may be less than the 50-year design lifetime time of the civil structure [3]. Therefore, maintenance must always be considered. For an example of life cycle analysis [17], the construction cost of using ECRs only involves a 20% increase because the price of epoxy coating reinforcement is 1.3 times that of BSRs [72]. However, patching and repair continuously adjust the net present value such that the ECR proposal is not competitive in the final comparison. 

A considerable discount is brought by stainless cladding technology. Basham [72] indicates that the unit cost of SCRs is 2.5 times that of BSRs, which is much more economical compared to the stainless bar [152]. Indeed, stainless steel is six to nine times more expensive than carbon steel [73]. Construction using SCRs is regarded as requiring no maintenance in a 100-year design lifetime [60,73,153]. 

**Figure 11:**
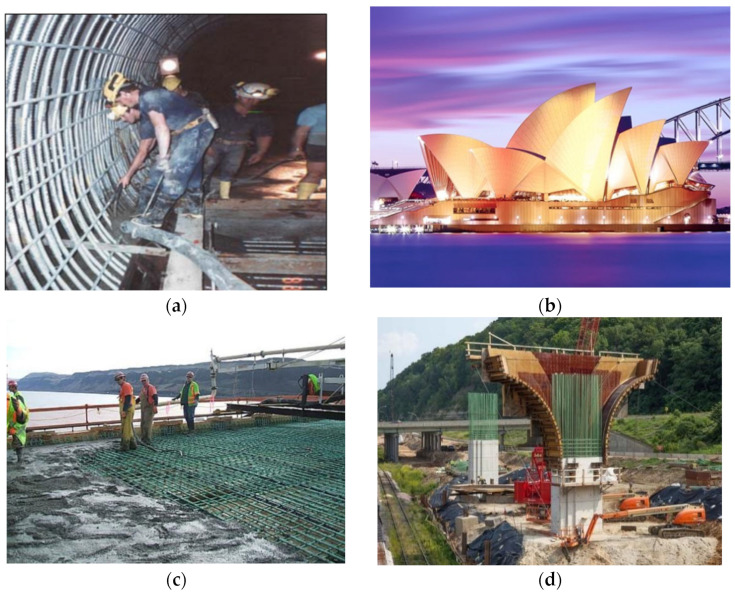

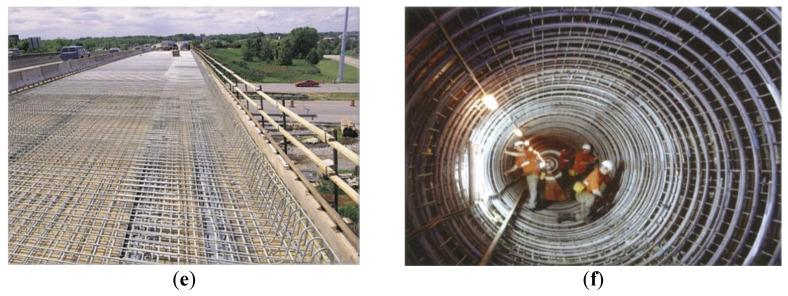
Applications of coated reinforcements: (**a**) Sewerage outfall tunnels, (**b**) Sydney Opera House, (**c**) The Biggs Rapids/Sam Hill Bridge (**d**) I-90 Dresbach Bridge Replacement (**e**) Ashland Avenue Bridge, (**f**) Hydro-Electric Power Station [16,43,155,159].

**Figure 12:**
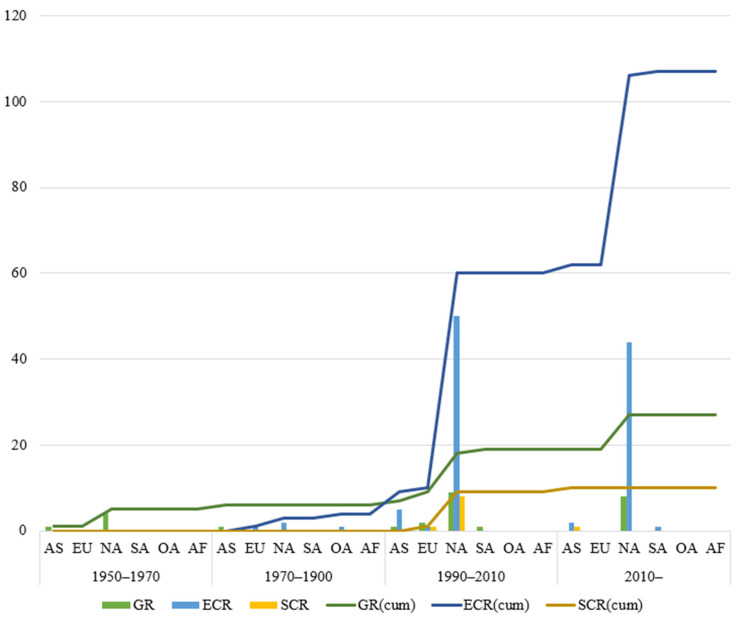
Applications of GR, ECR, SCR corresponding to the region and time Note: galvanized reinforcement (GR); epoxy coated reinforcement (ECR); stainless cladding reinforcement (SCR); Asia (AS); Europe (EU); North America (NA); South America (SA); Oceania (OA); Africa (AF).

Table 8 summarizes the applications of coated reinforcement, some images are shown in Figure 11. The data was collected from papers, company websites, and news. In general, most scenarios of using anti-corrosion reinforcement were marine infrastructures that require strict serviceability and have expensive maintenance costs. As demonstrated in Figure 12, the development of applications of GRs, ECRs, and SCRs is imbalanced in regions and time. There are considerable projects using anti-corrosion reinforcements in North America, but they are hard to find in Africa. Anti-corrosion reinforcements experienced a dramatic increase from 1990 to 2010. During this period, most projects were still built in North America, but Asian, European, and South American engineers also adopted anti-corrosion reinforcements. The development of society may affect the applications of anti-corrosion reinforcements. GRs have the earliest applications, followed by ECRs and SCRs. GRs have steadily increased in projects using anti-corrosion reinforcements. ECRs jumped fast after 1900, and their applications exceeded GRs after 1990. The mature manufacture and low cost of ECRs has promote their application. The number of projects using SCRs has remained approximately flat after the first increase from 1900 to 2010. Indeed, compared to GRs and ECRs, the relatively immature manufacture and high costs limit the popularity of SCRs. In 2015, the stainless-clad bimetallic steel bar (SCBSB) was produced by Hunan 3T New Materials CO., LTD [60]. The SCBSB improves the mechanical properties of SCRs and reduces the cost by approximately 30% [36,37,60,62,63,90,139]. A bay bridge in China adopted SCBSBs in 2021 [154]. As a relatively new material, the practical engineering application of SFCBs is rare.

**Table 8:** Application of coated reinforcements.

Reinforcement	Project	Year	Ref.	Figure
GR	The Longbird Bridge, Bermuda	1953	[155]	
GR	The Royal Bermuda Yacht Club, Bermuda	1968	[16]	
GR	The Egg, Albany, USA	1978	[155]	
GR	The new Watford Bridge, Bermuda, USA	1979	[16]	
GR	Sewerage outfall tunnels, Australia	-	[16]	Figure 11a
GR	Athens Bridge, Pennsylvania, USA	1953	[16]	
GR	Tioga Bridge, Pennsylvania, USA	1964	[16]	
GR	B776 pedestrian bridge, South Africa	1964	[155]	
GR	Ominichi Pier, Japan	-	[16]	
GR	Library Tower, Sydney, Australia	-	[16]	
GR	Deep Tunnel Sewage System, Singapore	-	[155]	
GR	Sydney Opera House, Sydney, Australia	1975	[155]	Figure 11b
GR	Parliament House, Australia	1983	[155]	
GR	Rome Mosque, Italy	1995	[155]	
GR	Sept-Ile Multi-Purpose Wharf Complex, Canada	2014	[155]	
ECR	Segmental bridge substructures in the Florida Keys, USA	1973	[28]	
ECR	Bridge decks located in Virginia, Wisconsin, Pennsylvania, New York, and Ohio, USA	1981	[28]	
ECR	Schieβberg Road Bridge in Leverkusen, Germany	1988	[156]	
ECR	12 km-long Belt Tunnel between Sprogø and Zealand in Denmark	1996	[56]	
ECR	Shantou LPG Wharf Engineering, China	1997	[157]	
ECR	Bachimen Bridge, China	2000	[158]	
ECR	Ministry of Defense building in Riyadh, Saudi Arabia	2003	[56]	
ECR	The Woodrow Wilson Bridge, Alexandria, USA	2006	[29]	
ECR	Museum of Islamic Art in Doha, USA	2008	[56]	
ECR	Alpha and Bravo Wharf Improvements (Marinas), Polaris Point, Guam	2008	[159]	
ECR	Richmond Olympic Speed Skating Oval, Canada	2008	[159]	
ECR	The Biggs Rapids/Sam Hill Bridge, USA	2009	[159]	Figure 11c
ECR	Bandra Worli Sea Link. India	2009	[29]	
ECR	US embassy in the Philippines, USA	2012	[56]	
ECR	Shijiazhuang–Wuhan passenger railway station in Henan, China	2012	[56]	
ECR	11th Street Bridge, Washington, USA	2013	[159]	
ECR	I-90 Dresbach Bridge Replacement	2016	[159]	Figure 11d
SCR	Three lane bridges, R12-4 of 33045, Lansing, Michigan, USA.	2001	[42]	
SCR	The bridge in I-94, Berrien County, Michigan, USA.	2008	[42]	
SCR	Highway 9 over South Holland canal—Ontario	2000	[43]	
SCR	Ashland avenue bridge—Brown County, Green Bay, Wisconsin	2001	[43]	Figure 11e
SCR	Hydro-Electric Power Station in Nant, Scotland—UK	2002	[43]	Figure 11f
SCR	Sturgeon River, Alberta	2002	[43]	
SCR	West Valley City interchange—East Bound, West Fargo, North Dakota	2001	[43]	
SCR	Putnam Road bridge, Shenectady County, N.Y.S.	2001	[43]	
SCR	Span A of B 635 of Route 460 in Campbell County, Virginia	2002	[43]	

Note: galvanized reinforcement (GR); epoxy coated reinforcement (ECR); stainless cladding reinforcement (SCR).

## 6. Future Works and Recommendations

Based on this review of previous studies and applications, recommended future research follows:

- Since GRs have been applied to constructions for many years, further studies are recommended to combine laboratory tests and field studies. This would help in precisely understanding the behaviors of GRs in concrete.
- ECRs have an inherent problem of permeability, which correlates to failure in corrosion resistance, as reported in early field studies. The corrosion performance of structures using ECRs needs continuous attention. Based on bibliometric analysis, this review finds that graphene oxide nanocomposite could improve the corrosion resistance of epoxy and enhance the performance of ECRs. This could be a possible research area.
- The corrosion mechanism of SCRs in concrete is a complicated subject. Current investigations study the corrosion behavior of SCRs by electrochemical corrosion. This is feasible but still different from the corrosion behavior of SCRs in concrete. Thus, more proper accelerated conditions need to be researched. In addition, investigation of the corroded bond strength of SCRs is rare, and future studies should fill this gap.
- The corrosion mechanism of SFCBs has not yet been individually investigated and the corrosion model of SFCBs in concrete is worth studying.
- Anti-corrosion reinforcements are used in infrastructures that may experience extreme loadings, such as fire, earthquake, storm and so on. Further experiments are recommended to couple corrosion with more loading scenarios.
- Anti-corrosion reinforcements are designed to meet requirements for the long-term use of infrastructures in an aggressive environment, which makes it necessary to conduct cost–benefit analysis. Thus, life-cycle cost analysis of structures using anti-corrosion reinforcements should be focused on.

## 7. Conclusions

This paper summarizes the existing knowledge on anti-corrosion reinforcements incorporating galvanized reinforcements (GRs), epoxy coated reinforcements (ECRs), stainless cladding reinforcements (SCRs), and steel-FRP composite bars (SFCBs) by providing a comprehensive review. The key aspects include manufacture, behaviors without corrosion, corrosion mechanism, and behaviors after corrosion. According to the presented investigations, the conclusions and research needs are drawn as follows:

*Influence of Manufacture*

- The heating process of producing GRs and ECRs can reduce their ductility, especially for coating cold-twisted steel bars.
- The rolling temperature may affect the strength of SCRs. With increase in rolling temperature, the promotion of strength is more distinct.
- The winding process has a negligible effect on the mechanical properties of SFCBs.
- GRs and ECRs should focus on minimizing the negative effect of manufacture on performance; while SCRs and SFCBs should reduce the cost and increase the production capacity.

*Behaviors without Corrosion*

- The tensile and fatigue behaviors of GRs and ECRs are mainly dominated by the steel substrate. They are expected to be as applicable to most projects as ordinary reinforcement.
- The tensile and fatigue behaviors of SCRs and SFCBs depend on the coating and core, and their interaction. SCRs show similar behaviors to BSRs because stainless steel and black steel have similar mechanical properties while they are metallurgically bonded, even after failure. SFCBs couple elastic–plastic steel and linear elastic FRP, which provides stable secondary stiffness. The force transfers between coating and core by friction.
- The bond strength of ECR and GR reinforced concrete was reported to be deficient. The performance of SCRs regarding bond strength is similar to that of BSRs. After surface treatment, SFCBs could have better performance than BSRs.

*Corrosion Mechanism*

- Due to cathodic protection, corrosion first consumes the galvanized layer and then the steel substrate.
- The steel substrate of ECR is usually first corroded because corrosive materials prefer to permeate the epoxy instead of decomposing.
- The corrosion of SCRs is complicate, combining electrochemistry with physics, and further research is needed.
- SFCBs have a similar corrosion mechanism to that of FRP bars, in which hydrolysis and sustained load can simultaneously contribute to the degradation of resin and fibers.

*Behaviors after Corrosion*

- Corrosion in galvanized layers has a negligible effect on the tensile and fatigue properties of GRs.
- SCRs have lower degradation of ductility in a given degree of corrosion compared to BSRs.
- The steel substrate of SFCBs was not corroded, and the degradation of SFCBs in corrosion is in ultimate strength instead of ductility.
- The fatigue properties of corroded SCRs are similar to those of corroded BSRs.
- GRs, ECRs, and SFCBs ensure better performance regarding bond strength than BSRs after corrosion.

*Applications of Anti-Corrosion Reinforcements*

- The advanced use of anti-corrosion reinforcements is uneven in regions, and may correlate to the development of technology and the economy.
- GRs and ECRs have considerable applications worldwide. With developments in their manufacture, SCRs may be an economical alternative to other anti-corrosion reinforcements. The application of SFCBs is rare.

## Figures and Tables

**Table 6:** Experiments on corroded fatigue properties of GR and SCR.

Ref	Type	Variable	Remarks
Hasegawa et al., 2017 [54]	GR	Humidity; Loading cycles	The humidity has a neglectable effect on the low cycle fatigue performance of galvanized reinforcements. When the number of cycles to fatigue exceeds 10^4^, the high humidity causes a reduction of fatigue strength. After failure, cracks on the interface were observed.
Hua et al., 2022 [139]	SCR	Corrosion degree; strain amplitudes	With the increase of corrosion degree and fatigue strain amplitude, the cycles of fatigue and total energy dissipation reduce. In the fatigue test, the deformation of stainless steel cladding and steel core are harmonious; no debonding was observed even in severe corrosion.

Note: galvanized reinforcement (GR); stainless cladding reinforcement (SCR).

## Abbreviations

BSR	black steel reinforcement
GR	galvanized reinforcement
ECR	epoxy coated reinforcement
SCR	stainless cladding reinforcement
FRP	fiber reinforced polymer
CFRP	carbon fiber reinforced polymer
GFRP	glass fiber reinforced polymer
BFRP	basalt fiber reinforced polymer
SFCB	steel-FRP composite bar
SCBSB	stainless-clad bimetallic steel bar
